# The impact of direct acting antivirals on hepatitis C virus disease burden and associated costs in four european countries

**DOI:** 10.1111/liv.14808

**Published:** 2021-02-24

**Authors:** Francesco S. Mennini, Andrea Marcellusi, Sarah Robbins Scott, Simona Montilla, Antonio Craxi, Maria Buti, Liana Gheorghe, Stephen Ryder, Loreta A. Kondili

**Affiliations:** ^1^ Economic Evaluation and HTA Centre for Economic and International Studies (EEHTA‐CEIS) Faculty of Economics University of Rome “Tor Vergata” Rome Italy; ^2^ Institute of Leadership and Management in Health Kingston Business School Kingston Univeristy London UK; ^3^ Department of Economic Strategy of Pharmaceutical Products Italian Medicines Agency Rome Italy; ^4^ Gastroenterology and Hepatology Unit Department of Internal Medicine and Medical Specialties "PROMISE" University of Palermo Palermo Italy; ^5^ Liver Unit Hospital Universitario Valle Hebron and CIBER‐EHD del Insitituto Carlos III Barcelona Spain; ^6^ Center for Digestive Diseases and Liver Transplantation Fundeni Clinical Institute University of Medicine and Pharmacy Carol Davila Bucharest Romania; ^7^ NIHR Nottingham Biomedical Research Centre Nottingham University Hospitals NHS Trust The University of Nottingham Nottingham UK; ^8^ Center for Global Health Istituto Superiore di Sanità Rome Italy

**Keywords:** break‐even, DAAs, HCV elimination, hepatitis C infection

## Abstract

**Background and Aims:**

We assessed the clinical and economic impact of direct‐acting antiviral (DAA) therapy for hepatitis C virus (HCV) in England, Italy, Romania and Spain.

**Methods:**

An HCV progression Markov model was developed considering DAA eligibility and population data during the years 2015‐2019. The period of time to recover the investment in DAAs was calculated as the cost saved by avoiding estimated clinical events for 1000 standardized treated patients. A delayed treatment scenario because of coronavirus disease (COVID‐19) was also developed.

**Results:**

The estimated number of avoided hepatocellular carcinoma, decompensated cirrhosis and liver transplantations over a 20‐year time horizon was: 1,057 in England; 1,221 in Italy; 1,211 in Romania; and 1,103 in Spain for patients treated during 2015‐2016 and 640 in England; 626 in Italy; 739 in Romania; and 643 in Spain for patients treated during 2017‐2019. The cost‐savings ranged from € 45 to € 275 million. The investment needed to expand access to DAAs in 2015‐2019 is estimated to be recovered in 6.5 years in England; 5.4 years in Italy; 6.7 years in Romania; and 4.5 years in Spain. A delay in treatment because of COVID‐19 will increase liver mortality in all countries.

**Conclusion:**

Direct‐acting antivirals have significant clinical benefits and can bring substantial cost‐savings over the next 20 years, reaching a Break‐even point in a short period of time. When pursuing an exit strategy from strict lockdown measures for COVID‐19, providing DAAs should remain high on the list of priorities in order to maintain HCV elimination efforts.

**Abbreviations**
DAA: direct‐acting antiviral
DC: decompenstated cirrhosis
DSA: deterministic sensitivity analysis
GDP: gross domestic product
HCC: hepatocellular carcinoma
HCV: hepatitis C virus
ILD: irreversible liver damage
LT: liver transplantation
PSA: probabilistic sensitivity analysis
SVR: sustained virologic response

**Key points**

- Despite the country‐specific dynamics and natural history of hepatitis C virus (HCV) infection in Italy, Spain, Romania, and England, expanding access to treatment will lead to a positive return on investment and is cost saving in <10 years.
- Not treating or delaying treatment of infected individuals will result in higher disease burden and costs, which could be avoided with immediate screening, linkage to care, and treatment of all HCV infected individuals.
- As lockdown orders for coronavirus disease 2019 (COVID‐19) begin to lift, providing direct acting antivirals (DAAs) should remain a priority for public health officials in order to maintain HCV elimination efforts.

## 1 INTRODUTION

Hepatitis C virus (HCV) is a leading cause of liver‐related morbidity and mortality worldwide.^1^, ^2^ The effect of antiviral therapy, in terms of the impact on clinical‐related outcomes rather than virological efficacy, changes over time according to the epidemiological profile of the disease, specific patient characteristics including fibrosis stage, and antiviral treatment efficacy.^3^, ^4^, ^5^, ^6^, ^7^ These factors vary by country, meaning country‐specific epidemiology of HCV infection, DAA treatment guidelines, and treatment access are expected to impact the burden of HCV‐liver related outcomes following viral eradication. No clinical studies have assessed the impact of antiviral therapy on long‐term morbidity and mortality, however, as it is unethical to maintain patients without therapy. Thus, only a modelling approach can address this issue and predict its impact on a population.^5^

In this study, we built country‐specific models using real‐life data of fibrosis stage, genotype distribution and treatment eligibility for England, Italy, Romania and Spain. These countries have varying epidemiological patterns of infection, treatment eligibility and gross domestic product (GDP), thus providing a comprehensive evaluation on how treatment access in these different landscapes can influence their clinical and economic burden of HCV. Additionally, for these countries, the costs associated with each state of liver disease was available in the literature. Based on these considerations, we could evaluate the impact of HCV‐related treatment policies on various economies throughout Europe.

The main objectives of this study were: (a) to estimate the number of HCV‐related liver disease cases avoided according to the modeled treatment scenarios in four European countries over a 20‐year time horizon; (b) to assess, by country, the impact of antiviral therapy on the direct cost of HCV‐related disease management; (c) to predict the neccessary time to recover the initial investment for treatment in each country; and (d) to evaluate how a treatment delay, because of the coronavirus disease 2019 (COVID‐19) pandemic, could impact disease burden outcomes.

## 2 MATERIALS AND METHODS

A country‐specific Markov model was designed to estimate the clinical and economic outcomes of expanded access to DAA therapy, considering the direct costs of HCV treatment in the European context (England, Italy, Romania and Spain). The model inputs are shown in Tables 1 and 2.

**TABLE 1:** Transition probabilities and efficacy of treatment (base‐case, deterministic and probabilistic sensitivity analysis parameters)

Annual probability of disease progression	Base‐case	Parameter value used in the DSA [11]	Standard error included in the PSA	Source
F0 to F1	0.117	0.113	**0,010**	[12]
F1 to F2	0.085	0.113	**0,007**	[12]
F2 to F3	0.120	0.113	**0,010**	[12]
F3 to F4	0.100	0.113	**0,009**	[13]
F4 to decompensated cirrhosis (DC)	0.030	0.041	**0,003**	[13]
F4 to HCC	0.050	0.021	**0,004**	[13]
Decompensated cirrhosis to HCC	0.100	0.014	**0,009**	[13]
Decompensated cirrhosis to transplant	0.110	0.031	**0,009**	[13]
HCC to transplant	0.200	0.031	**0,017**	[14]
SVR to HCC^a^	0.008	0.009	**0,000**	Assumption from [15]
SVR to transplant^a^	0.016	0.020	**0,002**	Assumption from [15]

Annual probability of progressing to death	Base‐case	[11]		Source
Decompensated cirrhosis to death (liver‐related)	0.090	0.221	**0,008**	[15]
HCC to death (liver‐related)	0.430	0.221	**0,037**	[14]
Transplant (procedure) to death (liver‐related)	0.150	0.1715	**0,013**	[14]
Transplant (following years) to death (liver‐related)	0.057	0.0353	**0,005**	[14]
Death from all other causes Italy				[16]
2015‐2016	0.064			Average age 61.84
2017‐2019	0.062			Average age 60.17
Death from all other causes Romania				Eurostat – Lifetable (2018) Table A1 [16]
2015‐2016	0.085			Average age 61.21
2017‐2019	0.085			Average age 61.21
Death from all other causes Spain				Eurostat – Lifetable (2018) Table A1 [16]
2015‐2016	0.049			Average age 53.97
2017‐2019	0.049			Average age 53.97
Death from all other causes UK				Eurostat – Lifetable (2018) Table A1 [16]
2015‐2016	0.051			Average age 52.67
2017‐2019	0.042			Average age 44.81

Efficacy of treatments—2015	Base‐case	Min	Max	Source
F0‐F3 to SVR (Genotype 1)	0.879	0.643	1.000	Appendix [17, 18]
F4‐DC to SVR (Genotype 1)	0.834	0.643	1.000	
F0‐F3 to SVR (Genotype 2)	0.742	0.500	1.000	Appendix [17, 18]
F4‐DC to SVR (Genotype 2)	0.742	0.500	1.000	
F0‐F3 to SVR (Genotype 3)	0.758	0.606	1.000	Appendix [17, 18]
F4‐DC to SVR (Genotype 3)	0.758	0.606	1.000	
F0‐F3 to SVR (Genotype 4 and more)	0.525	0.000	1.000	Assumed equal to F0‐F3
F4‐DC to SVR (Genotype 4 and more)	0.525	0.000	1.000	Appendix [17, 18]

Efficacy of treatments—2016	Base‐case	Min	Max	Source
F0‐F3 to SVR (Genotype 1)	0.983	0.963	1.000	Appendix [17]
F4‐DC to SVR (Genotype 1)	0.928	0.760	1.000	“
F0‐F3 to SVR (Genotype 2)	0.960	0.920	1.000	“
F4‐DC to SVR (Genotype 2)	0.952	0.710	1.000	“
F0‐F3 to SVR (Genotype 3)	0.960	0.940	1.000	“
F4‐DC to SVR (Genotype 3)	0.860	0.710	1.000	“
F0‐F3 to SVR (Genotype 4 and more)	0.963	0.940	1.000	“
F4‐DC to SVR (Genotype 4 and more)	0.943	0.800	1.000	“

Efficacy of treatments—2017‐2019	Base‐case	Min	Max	Source
F0‐F3 to SVR (Genotype 1)	0.980	0.784	1.000	Appendix [17]
F4‐DC to SVR (Genotype 1)	0.931	0.745	1.000	“
F0‐F3 to SVR (Genotype 2)	0.980	0.784	1.000	“
F4‐DC to SVR (Genotype 2)	0.970	0.776	1.000	“
F0‐F3 to SVR (Genotype 3)	0.950	0.760	1.000	“
F4‐DC to SVR (Genotype 3)	0.884	0.707	1.000	“
F0‐F3 to SVR (Genotype 4 and more)	0.970	0.776	1.000	“
F4‐DC to SVR (Genotype 4 and more)	0.961	0.769	1.000	“

Abbreviations: DC, decompensated cirrhosis; DSA, deterministic sensitivty analysis; HCC, hepatocellular carcinoma; ILD, irreversible liver damage; PSA, probabilistic sensitivity analysi; SVR, sustained virological response.

^a^ Only for SVR to ILD.

**TABLE 2:** Cost parameters (base‐case, deterministic and probabilistic sensitivity analysis parameters)

Cost of treatment	England				
	Base‐case	Min	Max	Standard error included in the PSA	Source
Treatment 2015	€ 30,000.00	€ 8,000.00	€ 30,000.00	€ 6,000.00	Assumption by local expert
Treatment 2016	€ 22,000.00	€ 8,000.00	€ 30,000.00	€ 4,400.00	“
Treatment 2017	€ 18,000.00	€ 8,000.00	€ 30,000.00	€ 3,600.00	“
Treatment 2018	€ 9,000.00	€ 8,000.00	€ 30,000.00	€ 1,800.00	“
Treatment 2019	€ 8,000.00	€ 8,000.00	€ 30,000.00	€ 1,600.00	

Other direct medical costs	Base‐case	Min	Max	SE	Source
F0	€ 202	€ 182	€ 223	€ 38	Appendix [24]
F1	€ 202	€ 182	€ 223	€ 38	“
F2	€ 1,051	€ 946	€ 1,156	€ 326	“
F3	€ 1,051	€ 946	€ 1,156	€ 47	“
F4	€ 1,668	€ 1,502	€ 1,835	€ 135	“
Decompensated Cirrhosis (DC)	€ 13,371	€ 12,034	€ 14,708	€ 1.932	“
HCC	€ 11,915	€ 10,723	€ 13,106	€ 3.470	“
Transplant (procedure)	€ 40,068	€ 36,061	€ 44,075	€ 19.241	“
Transplant (following years)	€ 3,214	€ 2,892	€ 3,535	€ 650	“
SVR	€ 715	€ 644	€ 787	€ 307	“
SVR to ILD states^a^	€ 1,668	€ 1,502	€ 1,835	€ 350	“

Cost of treatment	Italy				
	Base‐case	Min	Max	SE	Source
Treatment 2015	€ 25,000.00	€ 5,000.00	€ 25,000.00	€ 5,000.00	Assumption by local experts
Treatment 2016	€ 15,000.00	€ 5,000.00	€ 25,000.00	€ 3,000.00	“
Treatment 2017	€ 9,000.00	€ 5,000.00	€ 25,000.00	€ 1,800.00	“
Treatment 2018	€ 6,000.00	€ 5,000.00	€ 25,000.00	€ 1,200.00	—
Treatment 2019	€ 5,000.00	€ 5,000.00	€ 25,000.00	€ 1,000.00	

Other direct medical costs	Base‐case	Min	Max	SE	Source
F0	€ 234	€ 176	€ 292	€ 25	[8, 9, 10, 17]
F1	€ 234	€ 176	€ 292	€ 25	“
F2	€ 234	€ 176	€ 292	€ 25	“
F3	€ 617	€ 292	€ 942	€ 139	“
F4	€ 876	€ 397	€ 1,354	€ 205	“
Decompensated Cirrhosis (DC)	€ 6,627	€ 4,385	€ 8,868	€ 962	“
HCC	€ 12,896	€ 5,792	€ 20,000	€ 3.049	“
Transplant (procedure)	€ 73,774	€ 62,648	€ 84,900	€ 4.775	“
Transplant (following years)	€ 2,365	€ 0	€ 4,729	€ 1.015	“
SVR	€ 0	€ 0	€ 0	€ 0	“
SVR to ILD states^a^	€ 1,440	€ 397	€ 2,483	€ 448	Assumption from [25]

Cost of treatment	Romania				
	Base‐case	Min b	Max b	SE	Source
Treatment 2015	€ 15,000.00	€ 5,000.00	€ 15,000.00	€ 3,000.00	Assumption by local expert
Treatment 2016	€ 12,000.00	€ 5,000.00	€ 15,000.00	€ 3,000.00	“
Treatment 2017	€ 6,000.00	€ 5,000.00	€ 15,000.00	€ 2,400.00	“
Treatment 2018	€ 6,000.00	€ 5,000.00	€ 15,000.00	€ 1,200.00	“
Treatment 2019	€ 5,000.00	€ 5,000.00	€ 15,000.00	€ 1,000.00	

Other direct medical costs	Base‐case	Min	Max	SE	Source
F0	€ 77	€ 69	€ 85	€ 92	[26]
F1	€ 77	€ 69	€ 85	€ 92	“
F2	€ 77	€ 69	€ 85	€ 92	“
F3	€ 77	€ 69	€ 85	€ 371	“
F4	€ 582	€ 524	€ 640	€ 331	“
Decompensated Cirrhosis (DC)	€ 930	€ 837	€ 1,023	€ 3.407	“
HCC	€ 592	€ 533	€ 651	€ 8.330	“
Transplant (procedure)	€ 56,699	€ 51,029	€ 62,369	€ 12.103	“
Transplant (following years)	€ 6,892	€ 6,203	€ 7,581	€ 928	“
SVR	€ 0	€ 0	€ 0	€ 0	“
SVR from ILD states ^a^	€ 735	€ 662	€ 809	€ 750	“

Cost of treatment	Spain				
	Base‐case	Min	Max	SE	Source
Treatment 2015	€ 25,000.00	€ 5,000.00	€ 25,000.00	€ 5,000.00	Assumption by local expert
Treatment 2016	€ 15,000.00	€ 5,000.00	€ 25,000.00	€ 3,000.00	“
Treatment 2017	€ 9,000.00	€ 5,000.00	€ 25,000.00	€ 1,800.00	“
Treatment 2018	€ 6,730.00	€ 5,000.00	€ 25,000.00	€ 1,346.00	“
Treatment 2019	€ 5,000.00	€ 5,000.00	€ 25,000.00	€ 1,000.00	“

Other direct medical costs	Base‐case	Min	Max	SE	Source
F0	€ 306	€ 306	€ 306	€ 6	[27, 28]
F1	€ 306	€ 306	€ 306	€ 6	“
F2	€ 306	€ 306	€ 306	€ 6	“
F3	€ 306	€ 306	€ 306	€ 273	“
F4	€ 556	€ 556	€ 556	€ 342	“
Decompensated Cirrhosis (DC)	€ 2,266	€ 2,266	€ 2,266	€ 2.834	“
HCC	€ 8,630	€ 8,630	€ 8,630	€ 4.880	“
Transplant (procedure)	€ 121,707	€ 121,707	€ 121,707	€ 15.797	“
Transplant (following years)	€ 35,575	€ 35,575	€ 35,575	€ 13.238	“
SVR	€ 6	€ 6	€ 6	€ 2	[29]
SVR to ILD states ^a^	€ 162	€ 162	€ 162	€ 996	“

Abbreviations: DC, decompensated cirrhosis; HCC, hepatocellular carcinoma; ILD, irreversible liver damage; PSA, probabilistic sensitivity analysi; SVR, sustained virological response.

^a^ Weighted average of states F4, DC and HCC (HCC costs are assumed equal to those of DC).

### 2.1 Model structure

A published Markov model (Supporting Information Figure A1) capturing multiple states of morbidity and mortality^8^, ^9^, ^10^ and grounded in country‐specific parameters was used to evaluate HCV disease progression and related costs for 1000 standardized treated patients over a 20 year time horizon. The model structure considers 13 disease states (fibrosis stages from F0 to F4, decompensated cirrhosis (DC), hepatocellular carcinoma (HCC), first‐year transplant and subsequent years transplant, sustained virological response (SVR) from F0 to F3, SVR from irreversible liver damage (ILD), HCV‐related death, and death from other causes, and 41 transition probabilities.^8^, ^9^, ^10^ Events constituting advanced liver disease, such as ILD or DC, were considered as cumulative events in the model and not mutually exclusive. Two different time periods, which covered the evolution of DAA eligibility criteria and prioritization of patients in each country, were considered. The first period (2015‐2016) captures the time of treatment prioritization according to the severity of fibrosis stage (F0‐F4). In the the second time period (2017‐2019), all countries except Romania relaxed treatment restrictions, such that patients in earlier stages of fibrosis were eligible for treatment.

### 2.2 Transition probabilities

The probabilities of progressing through the various stages of disease were based on the results of a literature review (Table 1). Throughout annual cycles, patients could remain in their current liver disease stage or progress to a worse state according to the natural history of the disease. This progression can be stopped or slowed down by treatment. The DAA efficacy was expressed in terms of probability to reach an SVR state. If patients were cured in stages F0‐F3 (they move to the SVR state), the model assumed that liver damage was reversed. Patients achieving an SVR in stages F4, DC and HCC were no longer infectious, but they could incur additional liver damage (F4 and DC could progress to HCC, or need a LT). The probabilities of moving from SVR‐ILD to HCC or LT were weighted for the percentage of patients with compensated cirrhosis, DC and HCC. In the model, patients could die because of HCV‐related diseases only if they were in the DC, HCC, LT (procedure) or LT (following years) disease stages.^11^, ^12^, ^13^, ^14^, ^15^, ^16^, ^17^ As a certain proportion of patients with F3 fibrosis may in fact have undiagnosed F4 liver fibrosis (PITER data, not shown), 10% of patients that achieved an SVR in F3 were progressed in the model according to the transition probabilities of achieving SVR in stage F4.^18^

All transition probabilities are adjusted for competing probabilities of death from other causes according to the official data from each country (Supporting Information Table A1).^16^ For the HCC state, the probability of death because of HCV and the probability of transplant were assumed to be independent.

### 2.3 Epidemiological and clinical parameters

**FIGURE 1:**
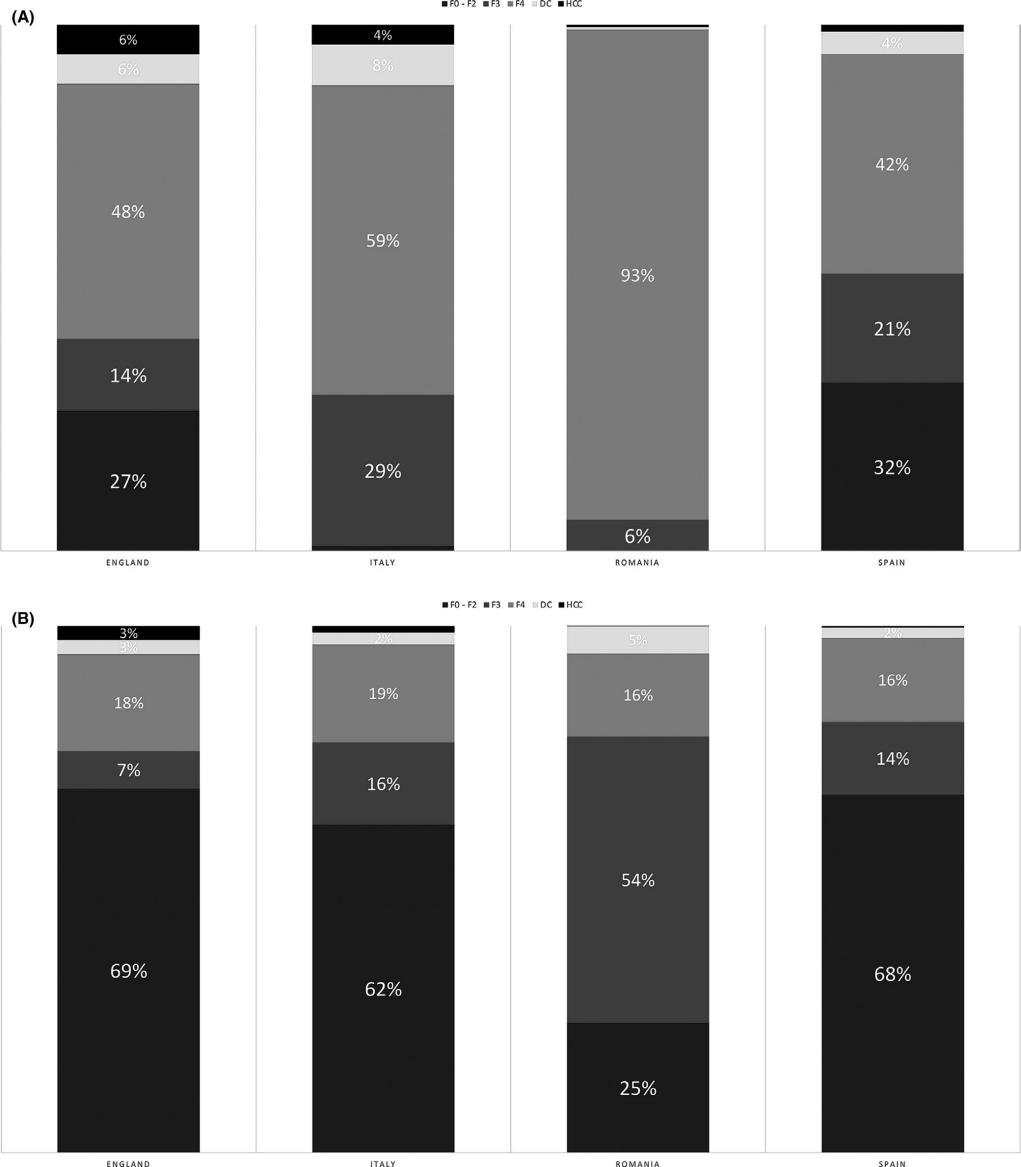
Proportion of patients by disease stage in England, Italy, Romania and Spain in 2015‐2016 and 2017‐2019. A, Period 2015‐2016; (B) Period 2017‐2019. F0‐F2: liver fibrosis stage F0‐F2; F3: liver fibrosis stage F3; F4: liver fibrosis stage F4 (liver cirrhosis); DC: Decompensated Cirrhosis; HCC: Hepatocellular Carcinoma

The model simulates a cohort of 1000 standardized treated patients according to real‐life fibrosis stage and genotype distribution data for England, Italy, Romania and Spain.^19^, ^20^, ^21^, ^22^, ^23^ Fibrosis stage by age and genotype distribution of treated patients by year for each of the countries included in the study were collected. The fibrosis distribution of treated patients for each country is reported in Figure 1, while the genotype distribution for each country during the two time periods evaluated is shown in Table A2 of the Supporting Information. According to these data, we simulated treatment in a cohort of standardized patients for each year evaluated, specifically years 2015‐2016 and another cohort of standardized patients treated during 2017‐1019. Avoided clinical events after therapy, potential cost reductions and return on investment of antiviral therapy were forecasted over a 20 year time horizon for both cohorts and outcomes were compared to a cohort of non‐treated patients.

### 2.4 Treatment efficacy

The efficacy of second‐generation DAA regimens used in each period was stratified by genotype distribution and the presence or absence of cirrhosis (F0‐F4, DC or HCC), as summarized in Table 1 and Tables A3‐A5 in the Supporting Information^3^, ^17^, ^18^.

### 2.5 Scenarios

The model simulates two different scenarios considering 1000 standardized patients over a 20‐year time horizon:

1. No treatment (base case scenario) : patients in disease stages F0‐F4 follow the natural history of HCV without any therapy;
2. Treatment scenarios: A cohort of 1000 standardized patients were treated in specific years according country specific genotype and fibrosis stage distribution, and followed over a 20 year time horizon after achieving SVR. This scenario was conducted for two cohorts of standardized patients. Scenario 2015‐2016 captured patients treated during 2015‐2016 according to the specific country's prioritized eligibility criteria and Scenario 2017‐2019, which captured patients treated during 2017‐2019 with no treatment restrictions or restrictions according to the eligibility criteria in Romania. The outcomes (clinical and economic) of both treatment scenarios were compared to the base case (no treatment) scenario.

Economic results were reported as the absolute difference between the estimated cost of the 2015‐2016 and 2017‐2019 scenarios to the “no treatment” scenario. The Break‐even analysis calculates the Break‐even Point in Time (BPT), which considers the time (in years) needed to minimize this difference to zero.

### 2.6 Economic parameters and analysis

Direct healthcare costs were those associated with the management of HCV‐related diseases, (including related outpatient visits, biochemical analyses, instrumental procedures, management and treatment of DC, HCC, and liver transplantation) along with costs of DAAs.^10^ The costs considered in the model estimate an average cost per health state considering treated/managed and not treated/not managed patients. All of the cost parameters considered for each country come from the cost‐effectiveness analysis that applies these costs with the same methodology applied in our case and are summarized in Table A6 of the Supporting Information. The average and range of costs (MINIMUM–MAXIMUM) of HCV‐related liver disease were derived from the literature (Table 2).^10^, ^17^, ^24^, ^29^ The average treatment cost of DAAs was estimated based on expert opinion and non‐official sources.

The associated per‐patient cost by disease stage post‐SVR was assumed to be null, presuming a state of full health after SVR for the patient until F3 fibrosis stage (Table 2 and Supporting Information Figure A1). The associated per‐patient cost by disease stage post‐SVR from ILD remained associated to the costs of ILD prior to therapy, independently of the achievement of SVR (Table 2) or the costs of other related events during disease progression. The annual costs saved were calculated as the estimated costs accrued of the no treatment scenario (base case) minus the estimated accrued costs of the two treatment scenarios (2015‐2016 and 2017‐2019 scenarios). Costs were expressed in Euros and were discounted at a rate of 3% annually.

### 2.7 Sensitivity analysis

To estimate the uncertainty of the economic results, probabilistic sensitivity analysis (PSA) and deterministic sensitivity analysis (DSA) were performed. The probabilistic distribution choice for cost was made by applying a gamma distribution and for epidemiological parameters, a beta distribution.^30^ Tables 1 and 2 report the standard error considered for the probabilistic distribution considering an arbitrary variation of 20%. Furthermore, 5000 Monte Carlo simulations were performed to provide 95% confidence intervals (95% CIs) for case and cost reduction at 20 years, Break‐even point, and case reduction at the Break‐even point. In the DSA, each sensible parameter of the model was subject to a variation derived from the literature (transition probabilities) or from an arbitrary variation^30^ as reported in Tables 1 and 2. The model results derived from each variation were compared to the value of the base case and represented by a tornado diagram.

### 2.8 Delayed treatment scenario

A delayed treatment scenario because of the COVID‐19 pandemic was developed to estimate the incremental cases associated with the subsequent scenario:

1. Base‐case 2020: all patients were treated during the first year and followed for 5 years;
2. Delay to treatment scenario: a delay in treatment for 3‐12 months was considered as a percentage of patients who may have their treatment postponed to the subsequent year.

The model was populated with the 2019 fibrosis distribution for each country, except for Spain where the fibrosis stage and genotype distributions considered in 2019 and 2020 are assumed the same as those reported in 2018 (Supporting Information Table A7). The liver related outcomes (number of irreversible liver damage (ILD) and number of liver‐related deaths standardized for a delay of treatment of 1000 patients over a period of 5 years were evaluated in the four countries. Preliminary estimates were reported for Italy and England in a previous Correspondence^31^

## 3 RESULTS

### 3.1 Characteristics of treated patients by country, according to fibrosis stage

As the four European countries have different treatment eligibility criteria, the distribution of fibrosis stages among treated patients varied significantly. Specifically, from 2015‐2016, 60% of patients in England, 70% of Italian patients, 94% of Romanian patients and 47% of Spanish patients had fibrosis stage F4 or more advanced decompensated cirrhosis or hepatocellular carcinoma. From 2017‐2019, there was a 30%‐60% increase in the number of F0‐F2 patients treated in England, Spain and Italy, but only a 25% increase in Romania. (Figure 1).

### 3.2 Evaluation of clinical outcomes from 2015‐2035

By expanding access to DAA therapies in 2017‐2019, the model estimated a decrease in end‐stage liver disease in all countries. There would be 640 fewer events of advanced liver disease in England, 626 fewer events in Italy, 643 fewer events in Spain and 739 fewer events in Romania over the next 20 years. More cases of ILD are avoided in 2015‐2016 as compared to 2017‐2019 (Table 3).

**TABLE 3:** Economic outcomes of expanding access to direct‐acting antiviral therapy over a 20‐year time horizon in England, Italy, Romania and Spain

	Year	Break even point in time (95% confidence interval)	Avoided cases (in BPT; 95% confidence interval)	Avoided cases (after 20 y; 95% confidence interval)	Avoided costs after 20 y (€ million; 95% confidence interval)
England	2015‐2016	7,8	667	1.057	€ 43,88
	(*Min‐Max*)	(*4,14*‐*12,59*)	(*534*‐*815*)	(*868*‐*1264*)	(*€ 17,52*‐*€ 82,13*)
	2017‐2019	5,8	250	640	€ 106,28
	(*Min‐Max*)	(*4,49*‐*7,27*)	(*215*‐*288*)	(*522*‐*770*)	(*€ 73,74*‐*€ 144,66*)
	Overall	6,5	405	820	€ 81,48
	(*Min‐Max*)	(*4,46*‐*8,92*)	(*335*‐*481*)	(*678*‐*975*)	(*€ 51,27*‐*€ 118,56*)
Italy	2015‐2016	7,7	797	1.221	€ 16,54
	(*Min‐Max*)	(*4,76*‐*11,34*)	(*625‐ 988*)	(*987*‐*1478*)	(*€ 0,55*‐*€ 58,74*)
	2017‐2019	4,4	179	626	€ 83,81
	(*Min‐Max*)	(*3,43*‐*5,49*)	(*147‐ 215*)	(*486*‐*783*)	(*€ 51,74*‐*€ 123,49*)
	Overall	5,4	383	886	€ 63,50
	(*Min‐Max*)	(*3,43*‐*7,81*)	(*291*‐*488*)	(*705*‐*1087*)	(*€ 30,41*‐*€ 108,57*)
Romania	2015‐2016	6,8	750	1.211	€ 45,29
	(*Min‐Max*)	(*3,74*‐*10,77*)	(*479*‐*1080*)	(*917*‐*1545*)	(*€ 3,15*‐*€ 142,08*)
	2017‐2019	6,4	337	739	€ 50,57
	(*Min‐Max*)	(*3,08*‐*10,91*)	(*191*‐*526*)	(*541*‐*967*)	(*€ 14,1*‐*€ 109,89*)
	Overall	6,7	510	€ 934	45,40
	(*Min‐Max*)	(*2,96*‐*11,94*)	(*291‐ 789*)	(*697*‐*1204*)	(*€ 7,68*‐*€ 115,98*)
Spain	2015‐2016	4,8	394	1.103	€ 289,63
	(*Min‐Max*)	(*3,36*‐*6,5*)	(*316‐ 479*)	(*903*‐*1323*)	(*€ 172,87*‐*€ 436,04*)
	2017‐2019	3,7	130	643	€ 241,50
	(*Min‐Max*)	(*2,68*‐*4,88*)	(*99*‐*166*)	(*509*‐*793*)	(*€ 146,9*‐*€ 359,24*)
	Overall	4,5	265	877	275,56
	(*Min‐Max*)	(*3,38*‐*5,77*)	(*213‐ 323*)	(*708*‐*1064*)	(*€ 170,9‐€ 404,83*)

Abbreviations: €, Euro; BPT, break‐even point in time; Min, minimum; Max, maximum

### 3.3 Evaluation of costs and return on investment from 2015‐2035

The potential reduction in clinical events over the next 20 years for patients treated between 2015‐2019 is cost saving in all countries. The overall savings for 1000 standardized treated patients over 20 years are estimated to be: about € 81 million in England, € 63 million in Italy, € 45 million in Romania and € 275 million in Spain.

**FIGURE 2:**
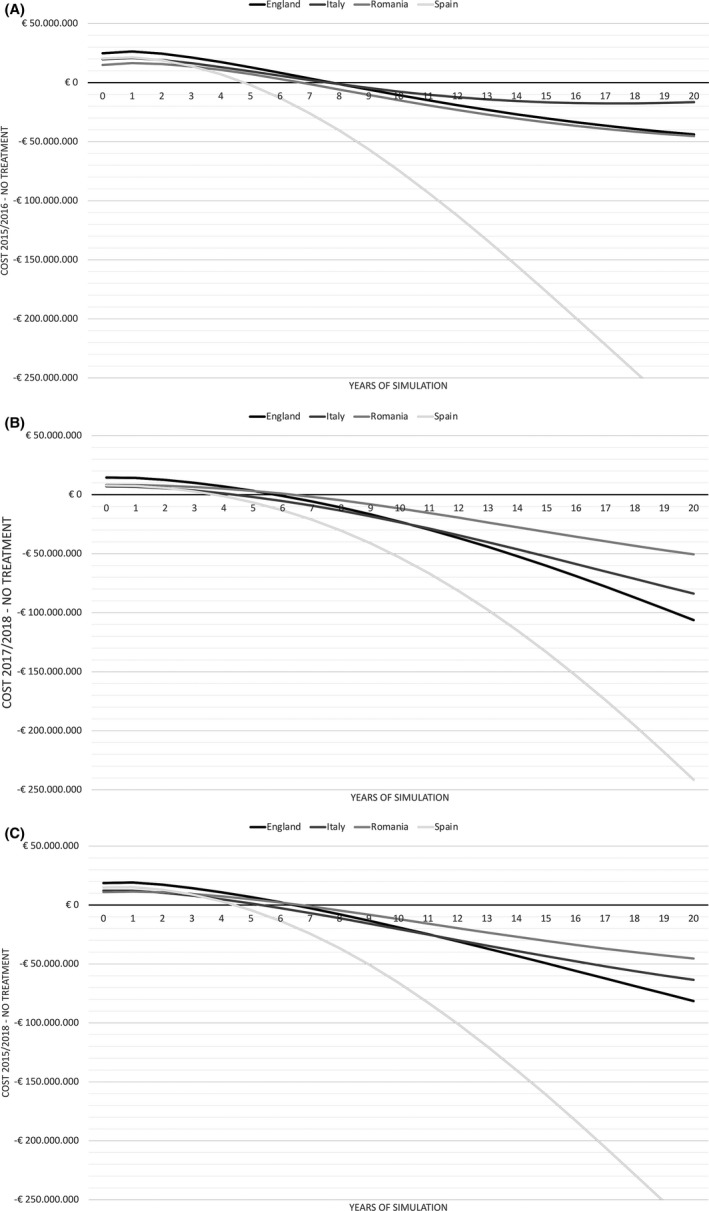
Differences in costs and the Break‐even Point in Time (BPT) by treatment scenario in England, Italy, Romania and Spain over a 20‐year time horizon. A, Period 2015‐2016; (B) Period 2015‐2016; (C) Overall Period 2015‐2019. The differences in costs are compared vs the No treatment scenario. The BPT is the point where where the each cost difference line crosses the abscissa (*X* axis)

In all countries, it would take less than 10 years to reach a Break‐ even point (Table 3). Overall, it would take England the longest amount of time to reach a Break‐ even point (6.5 years), while Spain would see the return on investment in <5 years (Figure 2).

**FIGURE 3:**
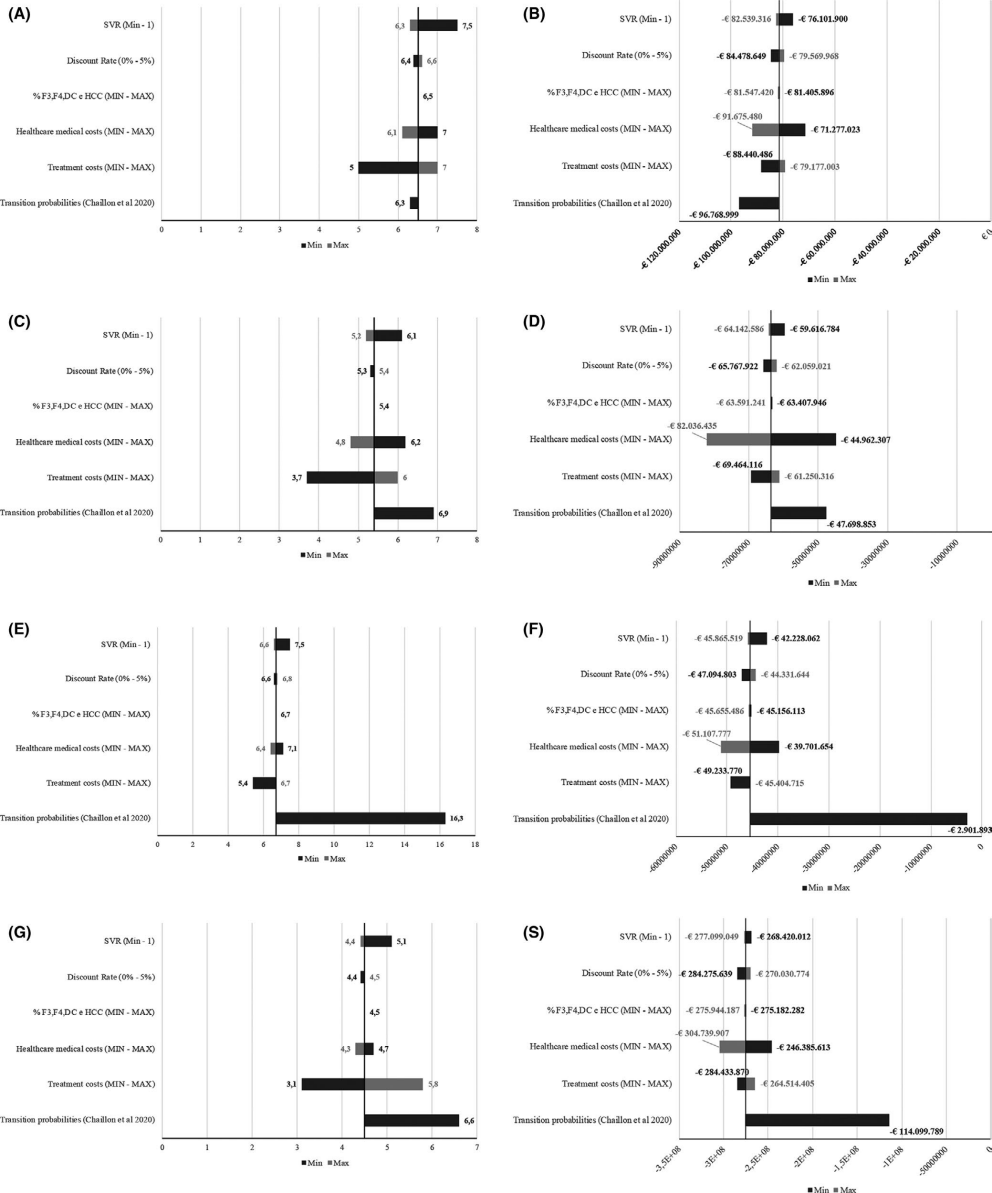
Deterministic Sensitivity Analysis assessing the impact of the parameters’ variation on the Break‐even estimations and total Costs avoided. A, England Break‐even estimations; (B) England costs avoided; (C) Italy Break‐even estimations; (D) Italy costs avoided; (E) Romania Break‐even estimations; (F) Romania costs avoided; (G) Spain Break‐even estimations; (S) Spain costs avoided

The costs avoided and Break‐even estimations over the next 20 years based on the DSA analysis are reported in Figure 3. Detailed results of DSA are reported in the Tables A5 and A6 and in Tables A8 and A9 of the Supporting Information. Transition probabilities had the highest impact on both the number of years needed until the Break‐even point and the total costs saved over the 20‐year time horizon for Italy, Spain, and Romania (25%, 59%, and 94%, respectively). For England, the variation of treatment cost led to a higher level of variability (−19% in the minimum scenario).

Considering the confidence intervals around the base case values (Table 3), which were estimated in the PSA analysis, the Break‐even points are similar to the base case values in all countries except Romania. In Romania, the max variation is almost double the time estimated in base case analysis. Higher variation is reported during the first treatment period (prioritized) vs the second treatment period (universal). The Break‐even point would take between 4.5 and 8.9 years in England, 3.4 years and 7.8 years in Italy, 3.0 and 12.0 years in Romania, and 3.4 and 5.8 years in Spain.

### 3.4 Delayed treatment because of COVID‐19

**FIGURE 4:**
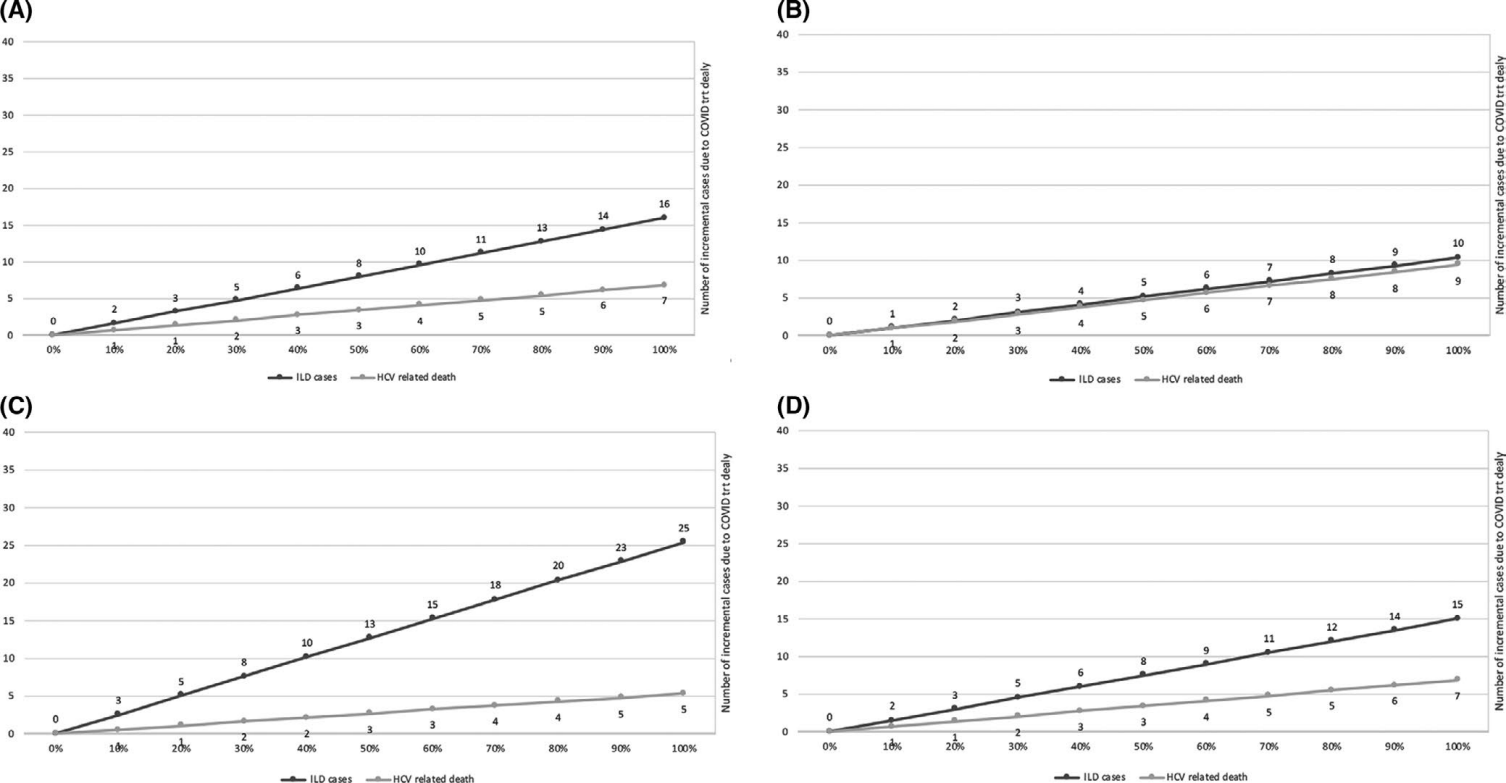
Additional cases of Irreversible Liver Disease and HCV‐related deaths because of treatment delay by COVID‐19 pandemic. Estimations for 1,000 patients at 5 years of follow‐up in England (A), Italy (B), Romania (C), Spain (D)

As shown in Figure 4, there will be a progressive increase in HCV disease‐related outcomes because of the delay in treatment caused by COVID‐19. Generally, there will be an increase in the number of estimated irreversibile liver disease cases for all countries. Romania would experience the largest increase in disease burden. Liver‐related deaths would increase at a slower rate compared to the increase in disease burden for all countries except Italy. In Italy, the additional number of liver related mortalities would increase at the same rate as the additional number of irreversibile liver disease cases.

## 4 DISCUSSION

The characteristics of HCV infection vary among countries because of different epidemic waves and modes of transmission. In Italy and Romania, an intensive epidemic wave occurred during the 1950s and 1960s, mainly associated with poor hygiene during invasive procedures, whereas in the other countries of this study, the most intensive epidemic waves occurred after the 1980s, mainly related to transfusions and intravenous drug use.^32^, ^33^, ^34^, ^35^, ^36^ Thus, considering these diverse waves and peak infection rates, differences are expected in the distribution of fibrosis stage cases and the impact of treatment on the natural history of infection.

The varying distribution of treated patients by disease stage in this study reflected the country‐specific treatment eligibility policies. In the 2015‐2016 time period, Italy and Romania were estimated to avoid the largest number of end‐stage liver disease events. Data have suggested that once cirrhosis is established, there is a high risk of developing HCC or hepatic decompensation and, following an episode of decompensation, there is a high risk of death.^37^, ^38^, ^39^, ^40^ This explains why countries which prioritized treatment of advanced fibrosis in 2015‐2016 (Italy and Romania) are estimated to avoid more late stage liver disease events compared to those with fewer treatment restrictions (England, Italy and Spain) in 2017‐2019.

In 2017‐2019, when fibrosis restrictions were removed in all countries except Romania, the time to return on investment was smaller compared to 2015‐2016. The different cost savings and consecutively the time to return of investment reflect, in part, the different costs of treating different disease stages in the four countries. Generally, the time to return on investment is related to two factors: cost of DAAs and liver disease costs. Specifically, the cost of DAAs administered to patients in 2015‐2016 was higher than that administered to patients in 2017‐2019, suggesting that the return on investment decreased over time was also because of the decreasing price of treatment. In a previous study in Italy in which patients treated in 2015 were evaluated using the same methodology, costs saved on avoided clinical events did not translate into a positive return on investment.^10^ This was because of the higher price of DAAs and also by treating patients with cirrhosis, which still accrued higher costs despite viral eradication.^41^

Generally, these estimations could vary given different progression rates. However, as seen by the sensitivity analysis results, a return of investment is expected within 10 years in all countries except Romania. If Romania expands DAA eligibility criteria, similar trends in costs saved and return on investment as seen in other countries, would be observed.

As COVID‐19 has become a global pandemic, we considered the impact that the disease may having on delaying access to DAA therapy. Despite major differences in HCV epidemiology and disease burden among the evaluated countries, the delayed treatment scenario generates comparable liver related mortality in the four countries evaluated. Over time, severe clinical outcomes because of delayed treatment will be evident in England, Spain and Romania, which will however result in less mortality than seen in Italy. In these three countries, while progressive liver disease will be evident across the next 5 years if treatment is delayed, the HCV infected population, as seen by the treatment data, is more likely to be in earlier stages of disease. On the contrary, in Italy, the liver morbidity is generally equal to the liver mortality because among individuals who are awaiting treatment and those who are expected to be diagnosed through screening in 2020‐2021, more than 100,000 individuals are estimated to have cirrhosis.^42^, ^43^ Hence, deferring DAA treatment for an additional six months in Italy would, over the next 5 years, lead to an additional 500 HCV‐related deaths. All of these deaths would be avoidable if testing and treatment were not deferred.^31^

If we accept the temporary need to reduce HCV elimination efforts because of the COVID‐19 pandemic, we should at least preserve the availability of immediate treatment for patients with advanced fibrosis or cirrhosis. Even if this may reduce severe disease outcomes, it will be only partially effective, as patients with early‐stage fibrosis may further progress and undiagnosed HCV patients with severe fibrosis will not be screened and treated in time. We suggest that in all countries worldwide, whatever the residual burden of untreated HCV infection, when pursuing an exit strategy from strict lockdown measures for COVID‐19, providing DAA treatments should remain high on the list of priorities in order to support HCV elimination strategies.^31^

Limitations to this modelling study exist. Firstly, several inputs of the model are based on literature data and applied to all countries. Drug costs were provided by country experts and are not official data. Thus, inputs may be over or underestimated for each country. However, despite the limited data available from the literature, particularly regarding the specific natural history of disease for each country, this approach considers all available evidence and represents a proxy of what decision‐makers could expect from their public health decisions. Second, antiviral treatment is considered as the disease cure if an SVR is reached. The SVR, however, primarily depends on the fibrosis stage at treatment onset as well as on host‐related and concomitant risk factors.^3^ Moreover, this modelling approach applied the same transition probabilities to the different pre‐treatment fibrosis stages, which may bias the outcomes. The transition probabilities used in this study are widely used in the literature, however, liver fibrosis progression is not a linear process. The progression probabilities could vary by country based on host and environmental factors, such as alcohol consumption, metabolic syndrome or comorbidities. These factors are equally distributed in different countries and may affect treatment outcomes and costs. In order to limit the effect of these uncertainties (and as commonly done in other modelling studies on the natural history of HCV infection), a sensitivity analysis was conducted, evaluating the impact of varying transition probabilities. Secondly, this analysis does not address how individuals will be identified for treatment, which is also cost intensive. A recent analysis determined that an expanded HCV screening stategy was cost effective in Italy, but the scenarios reported here analyse only the return on investment of antiviral therapy and does not consider screening costs.^42^ Thus, only efficacy and not effectiveness results in economic terms are reported in this analysis. Additional screening analyses in the three other countries included in this paper are warranted. Thirdly, this analysis was not focused on treatment as prevention, nor on the impact of reinfection of treated patients, particularly among people who inject drugs (PWID). Given the lack of available literature on the potential reinfection of treated individulas for each of the countries analysed, it was not possible to evaluate the role of new infections and reinfections on the return on investment of treated patients. This point should be considered in future studies on high risk populations, in which the probability of reinfection is high. In this particular study, data are based on the overall treated population and the impact of reinfection would consider only a portion of treated patients in each country.

In conclusion, the results of this study show that despite the country‐specific dynamics and natural history of HCV infection in Italy, Spain, Romania and England, there will be a positive return on investment and expanding access to treatment is cost saving in less than 10 years in the countries analysed. Universal access to therapies in all infected individuals will result in stronger economic returns and less disease burden. It should be noted though, as depicted in the delayed treatment scenario, that not treating or delaying treatment of infected individuals will result in higher disease burden and consecutively higher costs for the NHS. Policy makers could consider these efforts when determining the most cost‐effective methods for managing HCV infection across Europe. As lockdown measures for COVID‐19 begin to lift, providing DAAs should remain high on the list of priorities for public health officials in order to maintain HCV elimination efforts.

## CONFLICT OF INTEREST

Andrea Marcellusi, Sarah Robbins Scott and Simona Montilla have nothing to disclose; Francesco S. Mennini received advisor/speaker/research grants from Abbvie, MSD, Gilead, BMS; Antonio Craxì received advisor/speaker/research grants from AbbVie, Bayer, BMS, Gilead, Intercept and MSD. Liana Gheorghe received advisor/speaker/research grants from AbbVie, Bayer, Gilead, Merck, Takeda and Sanofi. Maria Buti received advisor/speaker/research grants from Abbvie and Gilead. Stephen Ryder received advisor/research grants from Abbvie and Gilead. Loreta A. Kondili received teaching grants from Abbvie and Gilead.

## Supporting information

Supplementary Material
