# Supplementary material for: The impact of direct acting antivirals on hepatitis C virus disease burden and associated costs in four european countries
Source: Liver Int. 2021 Feb 24;41(5):934–48. doi: 10.1111/liv.14808 (PMC8248004; doi:10.1111/liv.14808)
Supplement: Supplementary file 1 — Supplementary Material [file LIV-41-934-s001.docx]

# The Impact of Direct Acting Antivirals on Hepatitis C Virus Disease Burden and Associated Costs in Four European Countries

Francesco Saverio Mennini^1,2*^, Andrea Marcellusi^1,2*^, Sarah Robbins Scott^1^, Simona Montilla^3^, Antonio Craxi^4^, Maria Buti^5^, Liana Gheorghe^6^, Stephen Ryder^7^, Loreta A. Kondili^8^

***Francesco Saverio Mennini and Andrea Marcellusi equally contributed and should be considered as joint first authors.**

# Supplementary Material

**Figure A1 – Natural History of Hepatitis C Virus (HCV) Markov Model**


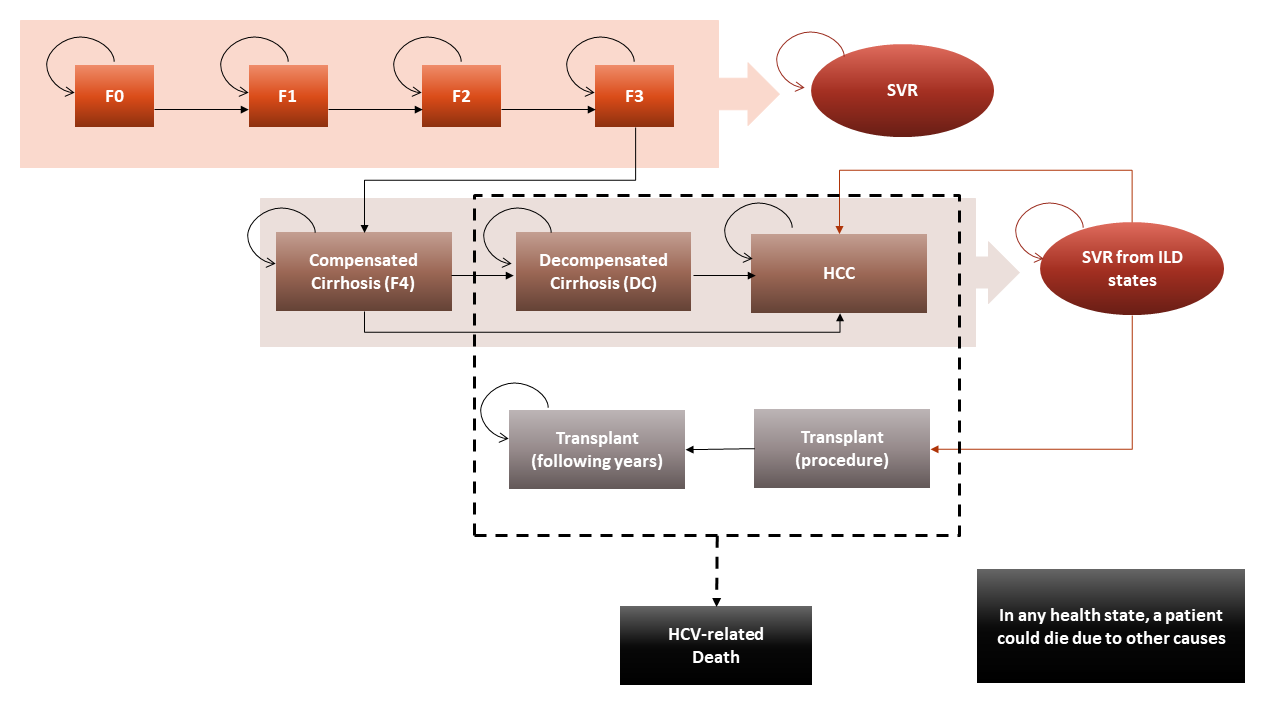


Legend: fibrosis stage; SVR: sustained virologic response; HCV: hepatitis C virus; DC: decompensated cirrhosis; HCC: hepatocellular carcinoma; ILD: irreversible liver damage

Progression of HCV liver disease was considered to increase with the severity of liver fibrosis (from F0 to F4 according to the Metavir classification) or progression to ILD stages.

For the F0-F4 and DC disease states, the probabilities of achieving SVR, disease progression, and HCV-related death were estimated.

| **Table A1 - Probability of dying between exact ages (age-specific mortality rate) 2018** | | |
| --- | --- | --- |
|  |  |  |

Source of data :

| **Eurostat (available in:** https://ec.europa.eu/eurostat/databrowser/view/demo_mlifetable/default/table?lang=en) | | | | | |
| --- | --- | --- | --- | --- | --- |
|  | | | | | |
| Age/Year | England | Italy | Spain | Romania |  |
| 45 | 0,00191 | 0,00112 | 0,00115 | 0,00352 |  |
| 46 | 0,00207 | 0,00130 | 0,00132 | 0,00387 |  |
| 47 | 0,00221 | 0,00144 | 0,00151 | 0,00418 |  |
| 48 | 0,00238 | 0,00154 | 0,00169 | 0,00475 |  |
| 49 | 0,00257 | 0,00171 | 0,00194 | 0,00518 |  |
| 50 | 0,00272 | 0,00195 | 0,00219 | 0,00662 |  |
| 51 | 0,00296 | 0,00210 | 0,00243 | 0,00584 |  |
| 52 | 0,00320 | 0,00233 | 0,00261 | 0,00719 |  |
| 53 | 0,00351 | 0,00251 | 0,00298 | 0,00809 |  |
| 54 | 0,00365 | 0,00288 | 0,00345 | 0,00891 |  |
| 55 | 0,00402 | 0,00308 | 0,00373 | 0,00993 |  |
| 56 | 0,00442 | 0,00335 | 0,00405 | 0,01032 |  |
| 57 | 0,00494 | 0,00372 | 0,00441 | 0,01128 |  |
| 58 | 0,00544 | 0,00422 | 0,00489 | 0,01246 |  |
| 59 | 0,00576 | 0,00456 | 0,00525 | 0,01308 |  |
| 60 | 0,00630 | 0,00514 | 0,00577 | 0,01499 |  |
| 61 | 0,00697 | 0,00535 | 0,00631 | 0,01570 |  |
| 62 | 0,00765 | 0,00602 | 0,00688 | 0,01644 |  |
| 63 | 0,00846 | 0,00670 | 0,00740 | 0,01765 |  |
| 64 | 0,00891 | 0,00718 | 0,00792 | 0,01780 |  |
| 65 | 0,01014 | 0,00794 | 0,00846 | 0,02042 |  |
| 66 | 0,01084 | 0,00870 | 0,00928 | 0,02141 |  |
| 67 | 0,01170 | 0,00966 | 0,01004 | 0,02313 |  |
| 68 | 0,01311 | 0,01022 | 0,01039 | 0,02377 |  |
| 69 | 0,01420 | 0,01150 | 0,01176 | 0,02604 |  |
| 70 | 0,01560 | 0,01260 | 0,01307 | 0,02729 |  |
| 71 | 0,01769 | 0,01432 | 0,01372 | 0,02954 |  |
| 72 | 0,01790 | 0,01548 | 0,01517 | 0,03188 |  |
| 73 | 0,02113 | 0,01708 | 0,01645 | 0,03553 |  |
| 74 | 0,02387 | 0,01950 | 0,01854 | 0,03997 |  |
| 75 | 0,02651 | 0,02164 | 0,02051 | 0,04232 |  |
| 76 | 0,02901 | 0,02369 | 0,02224 | 0,04768 |  |
| 77 | 0,03266 | 0,02673 | 0,02501 | 0,04906 |  |
| 78 | 0,03697 | 0,02945 | 0,02977 | 0,05761 |  |
| 79 | 0,04093 | 0,03351 | 0,03287 | 0,06313 |  |
| 80 | 0,04566 | 0,03821 | 0,03799 | 0,07104 |  |
| 81 | 0,05179 | 0,04301 | 0,04248 | 0,07902 |  |
| 82 | 0,05666 | 0,04963 | 0,04788 | 0,08772 |  |
| 83 | 0,06514 | 0,05608 | 0,05417 | 0,09844 |  |
| 84 | 0,07217 | 0,06541 | 0,06308 | 0,10729 |  |
| 85 | 1,00000 | 1,00000 | 1,00000 | 1,00000 |  |

In the base case analysis, we assumed that the mortality rate of people affected by chronic HCV infection in stages F0-F3 coincided with the mortality rate of the general population, as summarized above.

**Table A2– Genotype and fibrosis stage distribution by country**

| **Genotype Distribution England** | **2015 - 2016** | **2017 – 2019** | **Source** |
| --- | --- | --- | --- |
| G1 | 54% | 54% |  |
| G2 | 4% | 4% |  |
| G3 | 36% | 36% | **29,30** |
| G4 and other | 5% | 5% |  |
| **Genotype Distribution Italy** | **2015 - 2016** | **2017 – 2019** | **Source** |
| G1 | 64% | 58% |  |
| G2 | 15% | 21% | **31** |
| G3 | 14% | 14% |  |
| G4 and other | 8% | 8% |  |
| **Genotype Distribution Romania** | **2015 - 2016** | **2017 – 2019** | **Source** |
| G1 | 100% | 100% |  |
| G2 | 0% | 0% | **32** |
| G3 | 0% | 0% |  |
| G4 and other | 0% | 0% |  |
| **Genotype Distribution Spain** | **2015 - 2016** | **2017 – 2019** | **Source** |
| G1 | 77% | 67% |  |
| G2 | 3% | 4% | **33** |
| G3 | 9% | 14% |  |
| G4 and other | 12% | 15% |  |
| **Fibrosis Distribution England** | **2015 - 2016** | **2017 – 2019** | **Source** |
| F0 – F2 | 27% | 69% |  |
| F3 | 14% | 7% |  |
| F4 | 48% | 18% | **29,30** |
| DC | 6% | 3% |  |
| HCC | 6% | 3% |  |
| **Fibrosis Distribution Italy** | **2015 - 2016** | **2017 – 2019** | **Source** |
| F0 – F2 | 1% | 62% |  |
| F3 | 29% | 16% |  |
| F4 | 59% | 19% | **31** |
| DC | 8% | 2% |  |
| **HCC** | **4%** | **1%** |  |
| **Fibrosis Distribution Romania** | **2015 - 2016** | **2017 – 2019** | **Source** |
| F0 – F2 | 0% | 25% |  |
| F3 | 6% | 54% | **32** |
| F4 | 93% | 16% |  |
| DC | 0% | 5% |  |
| HCC | 0% | 0% |  |
| **Fibrosis Distribution Spain** | **2015 - 2016** | **2017 – 2019** | **Source** |
| F0 – F2 | 32% | 68% |  |
| F3 | 21% | 14% |  |
| F4 | 42% | 16% |  |
| DC | 4% | 2% | **33** |
| HCC | 1% | 0% |  |
|  |  |  |  |

**Table A3 - SVR rates by HCV RNA genotype and fibrosis stage used for estimating mean rates of SVR in 2015**

| Genotype 1 | | | | |
| --- | --- | --- | --- | --- |
| DAA | SVR for F0-F3 | SVR for F4 | SVR for DC | References |
| Sofosbuvir/Simeprevir/Ribavirin | 0.92 | 0.92 | 0.92 | [3] |
| Sofosbuvir/Simeprevir/Ribavirin | 0.95 | 0.95 | 0.95 | [3] |
| Sofosbuvir/ Ribavirin 1b | 0.64 | 0.64 | 0.64 | [3] |
| Sofosbuvir/ Ribavirin 1a | 0.67 | 0.67 | 0.67 | [3] |
| Sofosbuvir/Simeprevir | 0.95 | 0.88 | 0.88 | [4] |
| Sofosbuvir/Simeprevir | 0.96 | 0.94 | 0.94 | F0-F3 [4]; F4-DC [5]; |
| Sofosbuvir/Simeprevir | 0.97 | - | - | [4] |
| Sofosbuvir/Simeprevir | 0.97 | - | - | [6] |
| Mean | 0.88 | 0.83 | 0.83 |  |
| Genotype 2 | | | | |
| DAA | SVR for F0-F3 | SVR for F4 | SVR for DC | References |
| Sofosbuvir/ Ribavirin | 0.98 | 0.98 | 0.98 | [3] |
| Sofosbuvir/Simeprevir/Ribavirin | 0.5 | 0.5 | 0.5 | [3] |
| Mean | 0.74 | 0.74 | 0.74 |  |
| Genotype 3 | | | | |
| DAA | SVR for F0-F3 | SVR for F4 | SVR for DC | References |
| Sofosbuvir/ Ribavirin | 0.76 | 0.76 | 0.76 | [3] |
| Mean | 0.76 | 0.76 | 0.76 |  |
| Genotype 4 and others | | | | |
| DAA | SVR for F0-F3 | SVR for F4 | SVR for DC | References |
| Sofosbuvir/ Ribavirin | 0.63 | 0.63 | 0.63 | [3] |
| Sofosbuvir/Simeprevir/Ribavirin | 0.95 | 0.95 | 0.95 | [3] |
| Sofosbuvir/Simeprevir/Ribavirin | 0.00 | 0.00 | 0.00 | [3] |
| Mean | 0.53 | 0.53 | 0.53 |  |

DAA: direct acting antivirals; SVR: sustained virologic response; DC: decompensated cirrhosis

In order to estimate the mean sustained virologic response (SVR), the DAA regimens available and used in Europe in each year under evaluation (2015-2019) were considered. Specifically, the DAA regimens reported are those recommended as first line or alternative treatment by the European Association for the Study of the Liver (EASL) and American Association for the Study of Liver Diseases (AASLD) during the years considered in this study. These are provided in the organizations’ respective Guidelines on Treatment of HCV Chronic Infection for the years 2015 and 2016 [1,2].

**Table A4 - SVR rates by HCV RNA genotype and fibrosis stage used for estimating mean rates of SVR in 2016**

| Genotype 1 | | | | |
| --- | --- | --- | --- | --- |
| DAA | SVR for F0-F3 | SVR for F4 | SVR for DC | References |
| Daclatasvir/Sofosbuvir | 0.99 | 0.82 | 0.82 | F0-F3 [7]; F4 [8]; DC [9] |
| Daclatasvir/Sofosbuvir | 0.98 | - | 0.88 | F0-F3 [10]; DC [11] |
| Daclatasvir/Sofosbuvir | 1.00 | - | 0.80 | F0-F3 [10]; DC [11] |
| Paritaprevir/Ritonavir/Ombitasvir/Dasabuvir | 0.97 | 0.95 | - | F0-F3 [12]; F4 [13] |
| Paritaprevir/Ritonavir/Ombitasvir/Dasabuvir | 0.96 | 0.97 | - | F0-F3 [14]; F4 [13] |
| Paritaprevir/Ritonavir/Ombitasvir/Dasabuvir | 1.00 | 1.00 | - | F0-F3 [15]; F4 [13] |
| Ledipasvir/Sofosbuvir | - | 0.97 | 0.87 | F4 [16]; DC [17, 18] |
| Ledipasvir/Sofosbuvir | - | 0.97 | - | [19, 20] |
| Ledipasvir/Sofosbuvir | - | 1.00 | - | [21] |
| Daclatasvir/Sofosbuvir Gt1a | - | 0.90 | 0.76 | F4 [7]; DC [8] |
| Daclatasvir/Sofosbuvir Gt1b | - | 1.00 | 1.00 | F4 [7]; DC [8] |
| Daclatasvir/Sofosbuvir Gt1a | - | 0.76 |  | [8] |
| Daclatasvir/Sofosbuvir Gt1b | - | 1.00 |  | [8] |
| Mean | 0.98 | 0.94 | 0.86 |  |
| Genotype 2 | | | | |
| DAA | SVR for F0-F3 | SVR for F4 | SVR for DC | References |
| Daclatasvir/Sofosbuvir | 1.00 | 0.98 | 0.82 | F0-F4 [7]; DC [9] |
| Daclatasvir/Sofosbuvir | 0.92 | - | 0.88 | F0-F3 [10]; DC [11] |
| Daclatasvir/Sofosbuvir | - | - | 0.80 | [9] |
| Daclatasvir/Sofosbuvir | - | - | 0.71 | [9] |
| Daclatasvir/Sofosbuvir | - | - | 0.83 | [9] |
| Ledipasvir/Sofosbuvir | - | - | 0.87 | [17, 18] |
| Mean | 0.96 | 0.98 | 0.82 |  |
| Genotype 3 | | | | |
| DAA | SVR for F0-F3 | SVR for F4 | SVR for DC | References |
| Daclatasvir/Sofosbuvir | 0.97 | 0.89 | 0.82 | F0-F3 [22];  F4 [23]; DC [9] |
| Daclatasvir/Sofosbuvir | 0.94 | 0.86 | 0.88 | F0-F3 [22]; F4 [23]; DC [9] |
| Daclatasvir/Sofosbuvir | - | 0.86 | 0.8 | [11] |
| Daclatasvir/Sofosbuvir | - | 0.86 | 0.71 | F4 [11]; DC [9] |
| Daclatasvir/Sofosbuvir | - | - | 0.83 | [8] |
| Ledipasvir/Sofosbuvir | - | - | 0.87 | [17, 18] |
| Mean | 0.96 | 0.87 | 0.82 |  |
| Genotype 4 and others | | | | |
| DAA | SVR for F0-F3 | SVR for F4 | SVR for DC | References |
| Ledipasvir/Sofosbuvir | 0.95 | 0.95 | 0.87 | F0-F3 [24]; F4 [25]; DC [17, 18] |
| Paritaprevir/Ritonavir/Ombitasvir/Dasabuvir | 1 | 0.96 | - | F0-F3 [23]; F4 [26] |
| Paritaprevir/Ritonavir/Ombitasvir/Dasabuvir | 0.94 | 0.97 | - | [26] |
| Daclatasvir/Sofosbuvir | - | - | 0.82 | [9] |
| Daclatasvir/Sofosbuvir | - | - | 0.88 | [11] |
| Daclatasvir/Sofosbuvir | - | - | 0.8 | [9] |
| Mean | 0.96 | 0.96 | 0.84 |  |

DAA: direct acting antivirals; SVR: sustained virologic response; DC: decompensated cirrhosis

**Table A5 - SVR rates by HCV RNA genotype and fibrosis stage used for estimating mean rates of SVR in 2017-2019**

| Genotype | SVR for F0-F3* | SVR for F4* | SVR for DC* | References |
| --- | --- | --- | --- | --- |
| Genotype 1 | 0.98 | 0.94 | 0.88 | [27] |
| Genotype 2 | 0.98 | 0.99 | 0.85 | [27] |
| Genotype 3 | 0.95 | 0.89 | 0.85 | [27] |
| Genotype 4 and ohters | 0.97 | 0.98 | 0.88 | [27] |

*mean of treatments commonly used in Europe in 2017-2019

SVR: sustained virologic response; DC: decompensated cirrhosis

**Table A6–HCV-related liver disease cost parameters according to the fibrosis stage by country used in the model**

|  | England [35] | Italy [36] | Romania [37] | Spain [38] |
| --- | --- | --- | --- | --- |
| F0 | All values utilized within the model are consistent with a published systematic literature review, which have been used extensively in previous economic evaluations. All costs were inflated to 2013 values using the Hospital and Community Health Services index, where required. | By means of a systematic review of the available scientific literature, direct cost data associated with each disease were identified. The costs were actualized at 2013 and parametrized for comparison with the Price Index for ISTAT monetary revaluation. | Cost data include health care costs, drug costs, and indirect cost. Health care costs refer to the costs associated with the management of the disease and were extracted from two cost effectiveness studies from France and Romania. Health state costs in Romania were adjusted after discussion with clinical experts. All estimates reflect annual costs. | Medical consultations,  diagnostic tests, medication, etc. for monitoring the treatment and management of the disease and of the adverse effects that occurred |
| F1 |  |  |  |  |
| F2 |  |  |  |  |
| F3 |  |  |  |  |
| F4 |  |  |  |  |
| Decompensated Cirrhosis (DC) |  |  |  |  |
| HCC |  |  |  |  |
| Transplant (procedure) |  |  |  | Analytical data (including AFP), doppler ultrasound, spiral computed tomography scan, bone scintigraphy, surgical resection, percutaneous ethanol injection, orthotopic liver transplantation |
| Transplant (following years) |  |  |  |  |

**Table A7. Fibrosis stage and genotype distribution of patients treated with DAAs during the year 2019**

| Genotype | ENGLAND[29,30] | ITALY[31] | ROMANIA[32,33] | SPAIN[34] |
| --- | --- | --- | --- | --- |
| G1 | 54% | 58% | 100% | 67% |
| G2 | 4% | 21% | 0% | 4% |
| G3 | 36% | 14% | 0% | 14% |
| G4 other | 5% | 8% | 0% | 15% |
|  | 100% | 100% | 100% | 100% |
| Fibrosis stage | ENGLAND | ITALY | ROMANIA | SPAIN |
| F0 | 27% | 11% | 0% | 11% |
| F1 | 28% | 11% | 22% | 30% |
| F2 | 20% | 47% | 28% | 28% |
| F3 | 8% | 12% | 26% | 14% |
| F4 | 15% | 16% | 23% | 16% |
| DC | 2% | 2% | 0% | 2% |
| HCC | 0% | 2% | 0% | 0% |
| Total | 100% | 100% | 100% | 100% |

The year 2019 fibrosis stage distribution was considered for the “delayed treatment due to the COVID-19 pandemic” scenario.

**Table A8 – Model Outcomes Considering Deterministic Sensitivity Analysis Parameters (absolute value)**

This table illustrates the model outcomes for each one-way Deterministic Sensitivity Analysis performed. The parameters for the Deterministic Sensitivity Analysis are reported in Table 1.

|  | ENGLAND | | ITALY | | ROMANIA | | SPAIN | |
| --- | --- | --- | --- | --- | --- | --- | --- | --- |
| ***Tornado diagram (BEP)*** | ***Min°*** | ***Max^*** | ***Min°*** | ***Max^*** | ***Min°*** | ***Max^*** | ***Min°*** | ***Max^*** |
| Treatment costs (MIN - MAX) | 5.0 | 7.0 | 3.7 | 6 | 5.4 | 6.7 | 3.1 | 5.8 |
| Healthcare medical costs (MIN - MAX) | 7.0 | 6.1 | 6.2 | 4.8 | 7.1 | 6.4 | 4.7 | 4.3 |
| % F3,F4,DC e HCC (MIN - MAX) | 6.5 | 6.5 | 5.4 | 5.4 | 6.7 | 6.7 | 4.5 | 4.5 |
| Discount Rate (0% - 5%) | 6.4 | 6.6 | 5.3 | 5.4 | 6.6 | 6.8 | 4.4 | 4.5 |
| SVR (Min - 1) | 7.5 | 6.3 | 6.1 | 5.2 | 7.5 | 6.6 | 5.1 | 4.4 |
| Transition probabilities by Chaillon et al [28]. |  | ***6.3**** |  | 6.9* |  | ***16.3**** |  | ***6.6**** |
| ***Tornado diagram (Costs avoided)*** | ***Min°*** | ***Max^*** | ***Min°*** | ***Max^*** | ***Min°*** | ***Max^*** | ***Min°*** | ***Max^*** |
| Treatment costs (MIN - MAX) | -€ 88,440,486 | -€ 79,177,003 | -€ 69,464,116 | -€ 61,250,316 | -€ 49,233,770 | -€ 45,404,715 | -€ 284,433,870 | -€ 264,514,405 |
| Healthcare medical costs (MIN - MAX) | -€ 71,277,023 | -€ 91,675,480 | -€ 44,962,307 | -€ 82,036,435 | -€ 39,701,654 | -€ 51,107,777 | -€ 246,385,613 | -€ 304,739,907 |
| % F3,F4,DC e HCC (MIN - MAX) | -€ 81,405,896 | -€ 81,547,420 | -€ 63,407,946 | -€ 63,591,241 | -€ 45,156,113 | -€ 45,655,486 | -€ 275,182,282 | -€ 275,944,187 |
| Discount Rate (0% - 5%) | -€ 84,478,649 | -€ 79,569,968 | -€ 65,767,922 | -€ 62,059,021 | -€ 47,094,803 | -€ 44,331,644 | -€ 284,275,639 | -€ 270,030,774 |
| SVR (Min - 1) | -€ 76,101,900 | -€ 82,539,316 | -€ 59,616,784 | -€ 64,142,586 | -€ 42,228,062 | -€ 45,865,519 | -€ 268,420,012 | -€ 277,099,049 |
| Transition probabilities by Chaillon et al [28] |  | -€ 96,768,999* |  | -€ 47,698,853* |  | -€ 2,901,893* |  | -€ 114,099,789* |

*Model Outcome Obtained considering Challion et al [28].°Model Outcome Obtained considering Minimum Values ^Model Outcomes Obtained considering Maximum Values

**Table A9 – Deterministic Sensitivity Analysis Results: BEP and Costs Avoided (relative percentage difference vs base-case results)**

This table illustrates the increase or decrease model outcomes with respect to the base-case result for each one-way Deterministic Sensitivity Analysis performed. The parameters for the Deterministic Sensitivity Analysis are reported in Table 1.

|  |  |  | |  |  |  |  |  |  |
| --- | --- | --- | --- | --- | --- | --- | --- | --- | --- |
|  | ENGLAND | | ITALY | | | ROMANIA | | SPAIN | |
| ***Tornado diagram (BEP)*** | ***Min°*** | ***Max^*** | | ***Min°*** | ***Max^*** | ***Min°*** | ***Max^*** | ***Min°*** | ***Max^*** |
| Treatment costs (MIN - MAX) | -23.1% | 7.7% | | -31.5% | 11.1% | -19.4% | 0.0% | -31.1% | 28.9% |
| Healthcare medical costs (MIN - MAX) | 7.7% | -6.2% | | 14.8% | -11.1% | 6.0% | -4.5% | 4.4% | -4.4% |
| % F3,F4,DC e HCC (MIN - MAX) | 0.0% | 0.0% | | 0.0% | 0.0% | 0.0% | 0.0% | 0.0% | 0.0% |
| Discount Rate (0% - 5%) | -1.5% | 1.5% | | -1.9% | 0.0% | -1.5% | 1.5% | -2.2% | 0.0% |
| SVR (Min - 1) | 15.4% | -3.1% | | 13.0% | -3.7% | 11.9% | -1.5% | 13.3% | -2.2% |
| Transition probabilities (Chaillon et al) |  | ***-3.1%*** | |  | 27.8% |  | ***143.3%*** |  | ***46.7%*** |
| ***Tornado diagram (Costs avoided)*** | ***Min°*** | ***Max^*** | | ***Min°*** | ***Max^*** | ***Min°*** | ***Max^*** | ***Min°*** | ***Max^*** |
| Treatment costs (MIN - MAX) | 8.5% | -2.8% | | 9.4% | -3.5% | 8.4% | 0.0% | 3.2% | -4.0% |
| Healthcare medical costs (MIN - MAX) | -12.5% | 12.5% | | -29.2% | 29.2% | -12.6% | 12.6% | -10.6% | 10.6% |
| % F3,F4,DC e HCC (MIN - MAX) | -0.1% | 0.1% | | -0.1% | 0.1% | -0.5% | 0.6% | -0.1% | 0.1% |
| Discount Rate (0% - 5%) | 3.7% | -2.3% | | 3.6% | -2.3% | 3.7% | -2.4% | 3.2% | -2.0% |
| SVR (Min - 1) | -6.6% | 1.3% | | -6.1% | 1.0% | -7.0% | 1.0% | -2.6% | 0.6% |
| Transition probabilities (Chaillon et al) |  | ***18.8%*** | |  | -24.9% |  | ***-93.6%*** |  | ***-58.6%*** |

*Model Outcome Obtained considering Challion et al.

°Model Outcome Obtained considering Minimum Values

^Model Outcomes Obtained considering Maximum Values

DC: decompensated cirrhosis; HCC: hepatocellular carcinoma; SVR: sustained virologic response;

**References**

1. *EASL Recommendations on Treatment of Hepatitis C 2016.* J Hepatol, 2017. **66**(1): p. 153-194.
2. American Association for the Study of Liver Diseases (AASLD). *Recommendations for testing, Managing and Treating Hepatitis C*. 2016; Available from: http://hcvguidelines.org.
3. Kondili, L.A., et al., *Incidence of DAA failure and the clinical impact of retreatment in real-life patients treated in the advanced stage of liver disease: Interim evaluations from the PITER network.* PLoS One, 2017. **12**(10): p. e0185728.
4. Lawitz, E., et al., *Long-term follow-up of patients with chronic HCV infection following treatment with direct acting antiviral regimens: maintenance of SVR, persistence of resistance mutations and clinical outcomes.*, in *Paper presented at the European Association for the Study of the Liver meeting*. 2016 Apr 15: Barcelona, Spain.
5. Lawitz, E., et al., *Simeprevir plus sofosbuvir, with or without ribavirin, to treat chronic infection with hepatitis C virus genotype 1 in non-responders to pegylated interferon and ribavirin and treatment-naive patients: the COSMOS randomised study.* Lancet, 2014. **384**(9956): p. 1756-65.
6. Kwo, P., et al., *Simeprevir plus sofosbuvir (12 and 8 weeks) in hepatitis C virus genotype 1-infected patients without cirrhosis: OPTIMIST-1, a phase 3, randomized study.* Hepatology, 2016. **64**(2): p. 370-80.
7. Wyles, D.L., et al., *Daclatasvir plus Sofosbuvir for HCV in Patients Coinfected with HIV-1.* N Engl J Med, 2015. **373**(8): p. 714-25.
8. Poordad, F., et al., *Daclatasvir with sofosbuvir and ribavirin for hepatitis C virus infection with advanced cirrhosis or post-liver transplantation recurrence.* Hepatology, 2016. **63**(5): p. 1493-505.
9. Foster, G.R., et al., *Impact of direct acting antiviral therapy in patients with chronic hepatitis C and decompensated cirrhosis.* J Hepatol, 2016. **64**(6): p. 1224-31.
10. Sulkowski, M.S., et al., *Daclatasvir plus sofosbuvir for previously treated or untreated chronic HCV infection.* N Engl J Med, 2014. **370**(3): p. 211-21.
11. Leroy, V., et al., *Daclatasvir, sofosbuvir, and ribavirin for hepatitis C virus genotype 3 and advanced liver disease: A randomized phase III study (ALLY-3+).* Hepatology, 2016. **63**(5): p. 1430-41.
12. Ferenci, P., et al., *ABT-450/r-ombitasvir and dasabuvir with or without ribavirin for HCV.* N Engl J Med, 2014. **370**(21): p. 1983-92.
13. Poordad, F., et al., *ABT-450/r-ombitasvir and dasabuvir with ribavirin for hepatitis C with cirrhosis.* N Engl J Med, 2014. **370**(21): p. 1973-82.
14. Zeuzem, S., et al., *Retreatment of HCV with ABT-450/r-ombitasvir and dasabuvir with ribavirin.* N Engl J Med, 2014. **370**(17): p. 1604-14.
15. Andreone, P., et al., *ABT-450, ritonavir, ombitasvir, and dasabuvir achieves 97% and 100% sustained virologic response with or without ribavirin in treatment-experienced patients with HCV genotype 1b infection.* Gastroenterology, 2014. **147**(2): p. 359-365 e1.
16. Afdhal, N., et al., *Ledipasvir and sofosbuvir for untreated HCV genotype 1 infection.* N Engl J Med, 2014. **370**(20): p. 1889-98.
17. Charlton, M., et al., *Ledipasvir and Sofosbuvir Plus Ribavirin for Treatment of HCV Infection in Patients With Advanced Liver Disease.* Gastroenterology, 2015. **149**(3): p. 649-59.
18. Manns, M., et al., *Ledipasvir and sofosbuvir plus ribavirin in patients with genotype 1 or 4 hepatitis C virus infection and advanced liver disease: a multicentre, open-label, randomised, phase 2 trial.* Lancet Infect Dis, 2016. **16**(6): p. 685-697.
19. Bourliere, M., et al., *Ledipasvir-sofosbuvir with or without ribavirin to treat patients with HCV genotype 1 infection and cirrhosis non-responsive to previous protease-inhibitor therapy: a randomised, double-blind, phase 2 trial (SIRIUS).* Lancet Infect Dis, 2015. **15**(4): p. 397-404.
20. Reddy, K.R., et al., *Ledipasvir and sofosbuvir in patients with genotype 1 hepatitis C virus infection and compensated cirrhosis: An integrated safety and efficacy analysis.* Hepatology, 2015. **62**(1): p. 79-86.
21. Feld, J.J., et al., *Sofosbuvir and Velpatasvir for HCV Genotype 1, 2, 4, 5, and 6 Infection.* N Engl J Med, 2015. **373**(27): p. 2599-607.
22. Nelson, D.R., et al., *All-oral 12-week treatment with daclatasvir plus sofosbuvir in patients with hepatitis C virus genotype 3 infection: ALLY-3 phase III study.* Hepatology, 2015. **61**(4): p. 1127-35.
23. Hezode, C., et al., *Daclatasvir plus peginterferon alfa and ribavirin for treatment-naive chronic hepatitis C genotype 1 or 4 infection: a randomised study.* Gut, 2015. **64**(6): p. 948-56.
24. Kohli, A., et al., *Ledipasvir and sofosbuvir for hepatitis C genotype 4: a proof-of-concept, single-centre, open-label phase 2a cohort study.* Lancet Infect Dis, 2015. **15**(9): p. 1049-1054.
25. Abergel, A., et al., *Ledipasvir-sofosbuvir in patients with hepatitis C virus genotype 5 infection: an open-label, multicentre, single-arm, phase 2 study.* Lancet Infect Dis, 2016. **16**(4): p. 459-64.
26. Esmat, S.M., et al., *Efficacy of different modes of fractional CO2 laser in the treatment of primary cutaneous amyloidosis: A randomized clinical trial.* Lasers Surg Med, 2015. **47**(5): p. 388-95.
27. *Kondili, L.A., et al., Modeling cost-effectiveness and health gains of a "universal" versus "prioritized" hepatitis C virus treatment policy in a real-life cohort. Hepatology, 2017. 66(6): p. 1814-1825.*
28. *Chaillon*
29. National Institute of Health Research (NIHR), Nottingham Biomedical Research Centre, Public Health England’s (PHE) Hepatitis C in England 2018 report(https://assets.publishing.service.gov.uk/government/uploads/system/uploads/attachment_data/file/831155/Hepatitis_C_in_the_UK_2019_report.pdf
30. Local data from the NHS England Nottingham Hepatitis C operational Delivery Network ( https://www.england.nhs.uk/wp-content/uploads/2018/08/Operational-delivery-networks-for-hepatitis-C-care-adult.pdf)
31. Monitoraggio, U.R.d. Aggiornamentio dati Registri AIFA DAAs, epatitie C cronica. 2019, Agenzia Italiano del Farmaco.http://www.agenziafarmaco.gov.it/content/registri-farmaci-sottoposti-monitoraggio.
32. Therapeutic Protocol for Patients with HCV Chronic Hepatitis and Cirrhosis Treated with Interferon-free Direct-Acting Antivirals available in http://www.cnas.ro/media/pageFiles/Ordin%20nr.%201007_13.11.2019-criterii%20infectie%20cronica%20VHC%20la%20pacientii%20fara%20raspuns%20la%20AAD%20anterior.pdf
33. http://www.cnas.ro/media/pageFiles/Ordin%20nr.%20499_08.07.2015.pdf http://www.cnas.ro/media/pageFiles/ordin%2096221.11.2016_cvr%20ciroză%20hepatică%20decompensată_%20.pdf
34. **S**TRATEGIC PLAN FOR TACKLING HEPATITIS C IN THE SPANISH NATIONAL HEALTH SYSTEM https://www.mscbs.gob.es/ciudadanos/enfLesiones/enfTransmisibles/hepatitisC/PlanEstrategicoHEPATITISC/home.htm
35. *McEwan P, Webster S, Ward T et al. Estimating the cost-effectiveness of daclatasvir + sofosbuvir versus sofosbuvir + ribavirin for patients with genotype 3 hepatitis C virus. Cost Eff Resour Alloc. 2017;21;15:15.*
36. *Marcellusi A, Viti R, Capone A et al The economic burden of HCV-induced diseases in Italy. A probabilistic cost of illness model. Eur Rev Med Pharmacol Sci, 2015. 19:1610-20.*
37. *Ortsäter, G. Burden of hepatitis C in Europe—the case of France and Romania. Presented to: European Liver Patients Association, 2015*
38. *Buti M, Gros B, Oyagüez I et al. Cost-utility analysis of triple therapy with tela-previr in treatment-naïve hepatitis C patients. Farm Hosp.2014;38:418 29.*
